# Marine Compounds from the Far Eastern Organisms

**DOI:** 10.3390/md21020116

**Published:** 2023-02-09

**Authors:** Sergey A. Dyshlovoy, Timofey V. Malyarenko, Olesya I. Zhuravleva, Hiroshi Tomoda, Maxim E. Zhidkov

**Affiliations:** 1 Department of Oncology, Hematology and Bone Marrow Transplantation with Section Pneumology, Hubertus Wald-Tumorzentrum, University Medical Center Hamburg-Eppendorf, 20246 Hamburg, Germany; 2 Institute of High Technologies and Advanced Materials, Far Eastern Federal University, 690922 Vladivostok, Russia; 3 A.V. Zhirmunsky National Scientific Center of Marine Biology, Far Eastern Branch, Russian Academy of Sciences, 690041 Vladivostok, Russia; 4 G.B. Elyakov Pacific Institute of Bioorganic Chemistry, Far Eastern Branch, Russian Academy of Sciences, 690022 Vladivostok, Russia; 5 Drug Discovery Laboratory, Graduate School of Pharmaceutical Sciences, Kitasato University, Tokyo 108-8641, Japan

The term “Far East” implies a huge geographical region that consists of Eastern and Southeastern Asia, Eastern Russia and includes the waters of two oceans—the Pacific and Indian. Over 20 countries are considered a part of this region, many of which have access to the sea, and therefore, are actively involved in the research of the marine inhabitants and its metabolites. Compounds isolated from Far Eastern organisms are a significant and very important part of the whole pool of marine-derived substances—both new and previously known, bearing a unique chemical structure as well as an impressive spectrum of promising biological activities.

The simple search in Web of Knowledge (http://webofknowledge.com/, accessed on 30 January 2023) and PubMed databases (https://pubmed.ncbi.nlm.nih.gov/, accessed on 30 January 2023) results in a huge number of publications related to the natural compounds isolated from the marine organisms collected in the Far Eastern region. Interestingly, the number of reports on these compounds has started to grow significantly two decades ago, starting from 7 per year in 2000, up to 142 per year in 2020 (PubMed). This might be at least partially explained by the linguistic factors and more frequent and common use of the term “Far East” in scientific literature. However, a closer look indicates a strong correlation trend with the growing number of publications on marine compounds issued by the scientists affiliated in the countries located in the Far Eastern region. This might be explained well by the economic and technological progress made in the region over this time period, and therefore, the improved support of the studies regarding the local marine organisms’ metabolites.

The two marine-derived drugs which are already approved for clinical use have been developed based on molecules originally isolated from Far Eastern organisms. These drugs are an anticancer therapeutic, Halaven^®^, and an analgesic medication, Prialt^®^. Halaven^®^ was approved in 2010 for the treatment of metastatic breast cancer and further in 2016 for the treatment of liposarcoma [1]. This drug acts via an irreversible mitotic blockade [2]. Halaven^®^ is based on the Eribulin mesylate (E7389), which is a chemical derivative of polyether macrolide Halichondrin B, originally isolated from the Japanese marine sponge *Halichondria okadai* [3]. Prialt^®^ is an analgetic drug which was approved for the treatment of severe chronic pain in 2004 [4]. The active compound of this medication is a linear peptide ziconotide or ω-conotoxin MVIIA, of which specifically blocks N-type voltage-gated calcium channels. This leads to the inhibition of the release of pronociceptive neurotransmitters and neuromodulators, e.g., substance P, glutamate, and CGRP, thereby blocking a pain signal [4]. Additionally, several other compounds derived from the Far Eastern organisms (e.g. Plinabulin, Tetrodotoxin, etc.) are currently undergoing different phases of clinical trials [5].

The Special Issue “Marine Compounds from the Far Eastern Organisms” of *Marine Drugs* has covered the whole scope of the molecules, both novel and previously characterized, of which are isolated from the marine organisms inhabiting the Far Eastern region. This Special Issue is focused on the structure elucidation, chemistry, diversity, and various biological activities of these compounds. Thus, Tyrtyshnaia and colleagues have reported neuroprotective and neurogenetic activity of the previously known N-docosahexaenoylethanolamine (also known as DHEA or synaptamide) isolated form a squid *Berryteuthis magister* [6]. The authors used a rodent model with a sciatic nerve chronic constriction injury. Thus, the authors concluded that N-docosahexaenoylethanolamine may be of use in the therapy of neuropathic cognitive pain as well as emotional disorders [6]. Zhuravleva et al. reported an isolation of seven new compounds belonging to the family of deoxyisoaustamide from the Far Eastern marine fungus, *Penicillium dimorphosporum* KMM 4689 [7]. Some of these substances were shown to possess neuroprotective properties in the model of PQ(paraquat)-induced neurotoxicity in vitro [7]. Kvetkina and colleagues characterized peptide composition as well as antimicrobial, hemolytic, cytotoxic, and enzyme-inhibitory activities of extracts of five sea anemones harvested near Kuril and Commander Islands (Sea of Okhotsk and Bering sea, respectively) [8]. The authors showed that the extracts contain cytotoxic peptides that have a molecular weight of 4–6 kDa. The extracts were capable of killing Ehrlich carcinoma cells, they exhibited antibacterial properties, and had α-galactosidase inhibitory properties [8]. Girich et al. isolated and characterized new tripeptide compounds, asterripeptides A–C, which were found in marine fungus, *Aspergillus terreus* LM.5.2 [9]. These compounds exhibited moderate cytotoxic activity in human cancer cells as well as an ability to inhibit sortase A, suggesting, therefore, activity against *Staphylococcus aureus* [9]. Finally, Zhidkov and colleagues synthesized a small library of derivatives of the marine cytotoxic alkaloid fascaplysin [10]. The authors established a structural–activity correlation within this library and identified moieties which affected the cytotoxicity and selectivity of these compounds towards human prostate cancer cells in vitro. Thus, further directions of the structural optimization of fascaplysin were defined [10].

We sincerely thank all the authors who contributed to our Special Issue and are looking forward to further exciting discoveries.
